# Characterization of Phase Separated Planar Lipid Bilayer Membrane by Fluorescence Ratio Imaging and Scanning Probe Microscope

**DOI:** 10.3390/membranes12080770

**Published:** 2022-08-09

**Authors:** Yukihiro Okamoto, Kaito Hamaguchi, Mayo Watanabe, Nozomi Watanabe, Hiroshi Umakoshi

**Affiliations:** Division of Chemical Engineering, Graduate School of Engineering Science, Osaka University, 1-3 Machikaneyama-cho, Toyonaka, Osaka 560-8531, Japan

**Keywords:** nanodomain, scanning probe microscope, phase mode, solvatochromic fluorescence probe, supported lipid bilayer

## Abstract

The lipid membrane forms nanodomains (rafts) and shows heterogeneous properties. These nanodomains relate to significant roles in various cell functions, and thus the analysis of the nanodomains in phase-separated lipid membranes is important to clarify the function and role of the nanodomains. However, the lipid membrane possesses small-sized nanodomains and shows a small height difference between the nanodomains and their surroundings at certain lipid compositions. In addition, nanodomain analysis sometimes requires highly sensitive and expensive apparatus, such as a two-photon microscope. These have prevented the analysis by the conventional fluorescence microscope and by the topography of the scanning probe microscope (SPM), even though these are promising methods in macroscale and microscale analysis, respectively. Therefore, this study aimed to overcome these problems in nanodomain analysis. We successfully demonstrated that solvatochromic dye, LipiORDER, could analyze the phase state of the lipid membrane at the macroscale with low magnification lenses. Furthermore, we could prove that the phase mode of SPM was effective in the visualization of specific nanodomains by properties difference as well as topographic images of SPM. Hence, this combination method successfully gave much information on the phase state at the micro/macro scale, and thus this would be applied to the analysis of heterogeneous lipid membranes.

## 1. Introduction

The cell membrane plays a significant role in life events, such as barriers, molecular recognition, signal transduction, etc. [1] Thus, the development of its analysis method has been demanded at both the molecular and membrane level to reveal cell membrane functionality. The cell membrane is mainly comprised of lipids and membrane proteins [2], and shows a heterogeneous molecular distribution, a nanodomain, or a nanocluster, which is called a “raft” [3]. This “raft” also relates to various functions; in cell adhesion, rafts support tensile forces during cell migration by clustering adhesion molecules [4], and changing the shape of the cell [5]. In molecular interactions and reactions, rafts would enhance reaction rates and become superior reaction sites by enriching the membrane proteins and allowing effective binding between receptors and cofactors [6]. The rafts would also become chiral recognition sites because our group reported a chiral recognition mechanism by an artificial nanodomain that imitates the raft domain [7,8]. Hence, the analysis of the raft domain is significantly important in the research of cell membranes. 

However, raft analysis itself is very difficult because the size of the raft is too small (ca. 10–200 nm) and tremendous events occur in living cells during analysis. Thus, the artificial lipid membrane with phase separation, which imitates the cell membrane, has been adopted in the fundamental research of cell membranes. Lipid membranes with nanodomains can be prepared with the combination of higher and lower transition temperature lipid membranes [9]. By using these lipid membranes, planar or vesicle types, various methods have been applied for nanodomain analysis [10]. For example, fluorescence recovery after photobleaching (FRAP), fluorescence resonance energy transfer (FRET), fluorescence correlation spectroscopy (FCS), scanning probe microscope analysis (SPM), nuclear magnetic resonance (NMR), interference scattering microscopy, electron spin resonance spectroscopy (ESR), X-ray techniques, neutron scattering, etc. 

Among those, fluorescence analysis is one of the frequently employed methods in nanodomain analysis because the analysis is easily performed, and various fluorescence probes can be obtained and used in the analysis. The application of fluorescence probes indirectly informs the lipid membrane properties, such as fluidity [11,12], hydration degree [13], membrane potential [14], etc. Domain-specific partitioning probes [15] and different hydrophobicity probes [16] enable a site-specific analysis of lipid membranes. In addition, diffusion coefficients of proteins in and out of a nanodomain can be estimated by FRAP with fluorescence-labeled proteins [17]. Moreover, the existence of nanodomains and phase states (homogeneous or heterogeneous) can be investigated with FRET [18]. Though the fluorescence method shows utility in nanodomain analysis, it is impossible to directly observe and analyze both the nanodomain and its surroundings because lipids do not show any fluorescence, and a nanodomain size is a few hundred nanometers, which is below the diffraction limit of a microscope [19]. Moreover, especially, the analysis of supported lipid bilayer (SLB) with fluorescence probes faces the problem of photodegradation, and in some cases, requires a highly sensitive apparatus and a large number of probes. This excess number of probes would cause a change in membrane structures and properties [20]. 

On the other hand, SPM is one of the most powerful methods to directly visualize and analyze surface topologies and properties of nanomaterials, such as magnetic, viscoelastic, molecular interactions [21,22], etc. The nanodomain in a lipid membrane shows a ca. 10–100 nm size and a ca. 0.5–1.5 nm height difference between the nanodomain and its surroundings (liquid-ordered: L_o_ and liquid-disordered: L_d_) [23,24]. In addition to topological differences, property differences would be observed, i.e., the viscoelasticity [22], lipid packing degree [25], molecular interaction [26], etc. Therefore, the application of SPM enables direct visualization and analysis of nanodomains and their surroundings [27], while SPM is difficult to apply to whole membrane analysis. 

From the above information, the combination of fluorescence and SPM analysis can inform the macro (large area of membrane) and micro (specific parts of membrane) properties and would be a powerful method in raft (nanodomain) analysis. In this paper, we aimed to prove the utility of these improved combination methods with the phase-separated SLBs as a cell membrane model. By using solvatochromic fluorescence probes, LipiORDER, SLB’s polarity (hydration degree) and phase state in the macroscale were successfully analyzed with a lower magnification lens, which could not be achieved with Laurdan. Subsequently, the domain size, morphology, and type were analyzed by the SPM. The combination of different modes in the SPM enabled the microanalysis of nanodomains in detail. Especially, nanodomains were clearly visualized by the phase mode of the SPM as surface property differences, even if the topological profile was unclear. Therefore, we successfully demonstrated that this combination method would be a powerful tool for nanodomain (raft domain) analysis.

## 2. Materials and Methods

### 2.1. Materials

A 1-palmitoyl-2-oleoyl-glycero-3-phosphocholine (POPC), 1 2-dipalmitoyl-*sn*-glycero-3-phosphocholine (DPPC), and egg sphingomyelin (SM) (fatty acid distribution: 16:0(86%), 18:0 (6%), 22:0(3%), 24:1(3%), unknown (2%)) were purchased from Avanti Polar Lipids, Inc. (Alabaster, AL, USA). Cholesterol (CHOL) was purchased from Sigma-Aldrich (Sigma-Aldrich Co. LLC, St. Louis, MO, USA). Sodium dihydrogen phosphate, disodium hydrogen phosphate, and concentrated sulfuric acid were obtained from Kanto Chemical Co., Inc. (Tokyo, Japan). Chloroform, hydrogen peroxide, and 6-Lauroyl-2-dimethylamino naphthalene (Laurdan) were obtained from Wako Pure Chemicals Industries, Ltd. (Osaka, Japan). The stock solution of Laurdan (1 mM) was prepared with ethanol. Ultra-pure water was prepared with Direct-Q UV 3 (Merck, Osaka, Japan). Phosphate buffer (PB) was prepared with sodium dihydrogen phosphate and disodium hydrogen phosphate. LipiORDER was purchased from Funakoshi Co., Ltd. (Tokyo, Japan), and its stock solution (3.0 mM) was prepared with dimethyl sulfoxide (Kanto Chemical Co., Inc., Tokyo, Japan). Piranha solution was prepared from concentrated sulfuric acid and hydrogen peroxide, with a volumetric ratio of 3:1. All reagents were used without further purification. Cover glass (18 mm × 18 mm) with a thickness of 0.12–0.17 mm, and 35 mm diameter glass-bottom dishes were obtained from Matsunami (Osaka, Japan).

### 2.2. Preparation of Small Unilamellar Vesicles (SUVs) 

Liposomes with 50 nm diameter (molar ratio (%) of POPC: SM: CHOL = 20:40:40, 37:32:31, 49:29:22, 60:26:14, and 100:0:0, DPPC = 100) were prepared using a freeze-thaw extrusion method as follows: The chloroform solution of lipids was dried in a round-bottomed flask under vacuum with a rotary evaporator to prepare a lipid thin film. The thin film was hydrated with 25 mM PB (pH = 7.5) at room temperature for vesicle suspension. The vesicle suspension was frozen at −80 °C and thawed at 60 °C to enhance the transformation of small vesicles into large multilamellar vesicles. This freeze-thaw cycle was performed five times. The multilamellar vesicles were used to prepare small unilamellar vesicles (SUVs) by extruding the multilamellar vesicles suspension 13 times through two layers of polycarbonate membranes, with mean pore diameters of 50 nm (Avestine Inc., Ottawa, ON, Canada), using an extruding device (Liposofast; Avestine Inc., Ottawa, ON, Canada). The obtained 50 nm SUVs were suspended in 25 mM PB (pH = 7.5) (final lipid concentration: 1.0 mg/mL). 

### 2.3. Preparation of Supported Lipid Bilayer (SLB) on the Cover Glass 

SLB on cover glass was prepared according to the reported paper [28]. A cover glass was cleaned with piranha solution for over 12 h and then washed with ultra-pure water, annealed at 450 °C (FO100, Yamato Scientific Co., Ltd., Tokyo, Japan) for 2 h, and then cooled in a desiccator. LIQUID BLOCKER (Daido Sangyo Co., Ltd., Tokyo, Japan) was used for hydrophobic barrier formation to prevent liposome suspension from flowing out. After the evaporation of LIQUID BLOCKER’s solvent, 300 μL of liposome suspension was poured into this cover glass. This cover glass was incubated for 1 h at 60 ℃ in an incubator (CIW-450, AS ONE Corporation, Osaka, Japan), and then washed with ultra-pure water to remove immobilized liposomes. In preparation for SLB stained with LipiORDER, SUVs were stained with LipiORDER as follows: one μL of LipiORDER (3.0 mM) was mixed with 1 mL of SUVs (lipid concentration: 1.0 mg/mL) and this suspension was incubated for 30 min in the dark. Other procedures for SLB stained with LipiORDER were the same as non-stained SLB preparation. In the case of the photostability test, DPPC SLB was prepared under the same procedures and following conditions; Final concentration of LipiORDER, Laurdan, and 50 nm DPPC vesicles were 3.1 µM, 10 µM, and 1.0 mM, respectively. 

### 2.4. Characterization of (POPC/SM/CHOL) Small Unilamellar Vesicles with LipiORDER

The polarity and phase states of 50 nm SUVs were analyzed with LipiORDER. The final concentrations of lipid and LipiORDER were adjusted to 1.0 mg/mL and 3.0 µM, respectively. The fluorescence spectra of LipiORDER excited at 405 nm were measured from 450 nm to 650 nm with a fluorescence spectrophotometer FP-6500 (JASCO, Tokyo, Japan). Polarity and phase state of SUVs were evaluated with a fluorescence intensity ratio (*F*_575_/*F*_510_), which is defined as follows:

*F*_575_/*F*_510_ = (Fluorescence intensity at 575 nm)/(Fluorescence intensity at 510 nm) (1)

### 2.5. Observation and Analysis of Supported Lipid Bilayer (SLB) with a Fluorescence Microscope and Scanning Probe Microscope

An SLB was immersed in background solution (ultra-pure water) in the glass bottom dish and mounted on a fluorescence microscope (BX53, Olympus, Tokyo, Japan). This fluorescence microscope was equipped with ×10 lenses, a mercury lamp (130 W, U-HGLGPS, Olympus, Tokyo, Japan), filters (U-FUW for Laurdan, excitation filters: 340–390 nm, dichroic mirror: 410 nm, and barrier filter: 420 nm~; for LipiORDER, excitation filters: 389/38 nm, dichroic mirror: 414 nm, and barrier filter: 450 nm~), a complementary metal oxide semiconductor (CMOS) camera (ORCA-Fusion, HAMAMATSU photonics, Japan), and image splitting optics (W-VIEW GEMINI A12801-01, HAMAMATSU photonics, Japan) containing the following filter sets: dichroic mirror: 560 nm and barrier filters: 510/84 nm and 574 nm~. In addition, the thermal plate for temperature management at 25 °C (Tokai Hit., Co., Ltd, Shizuoka, Japan) was mounted on the stage of a fluorescence microscope, and a personal computer was connected to the CMOS camera for data analysis. The fluorescence images from dual-wavelength and the fluorescence intensity ratio (*F*_574~_ from 574 nm~/*F*_510_ from 510/84 nm) images were obtained and analyzed with cellSens Dimension (CS-DI5-SET, Olympus, Tokyo, Japan) after the removal of background fluorescence. The background fluorescence signal was defined as the fluorescence signal from the glass parts in the SLB, which was prepared by scratching the SLB. Similarly, in the photostability test, the fluorescence intensity data from the SLB was used after subtraction of the background signal, and the normalized intensity was plotted. Optical power irradiated to the LipiORDER stained SLB was measured at 400 nm with an optical power meter (PM160, Thorlabs) and ×10 water immersion lens (NA: 0.3), and its value was 87 mW.

Scanning probe microscope (SPM) analysis was conducted with the scanning probe microscope SPM-9700HT (Shimadzu, Kyoto, Japan). Operation conditions were as follows: dynamic mode, phase mode, and cantilevers (OMCL-AC200TS, Olympus, Tokyo, Japan) with a spring constant of 9 N/m. 

## 3. Results and Discussion

**Figure 1:**
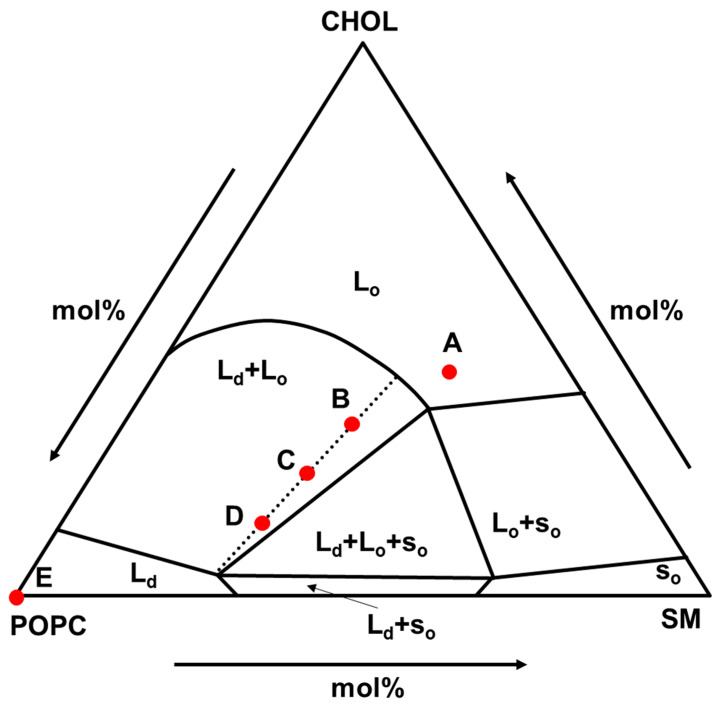
Phase diagram of POPC/SM/CHOL multilamellar vesicles at 23 °C reported by [30]. Mole ratio (%) of lipid composition (POPC/SM/CHOL): (A) (20/40/40), (B) (37/32/31), (C) (49/29/22), (D) (60/26/14), and (E) (100/0/0). s_o_: gel phase, L_o_: liquid-ordered phase, L_d_: liquid disordered phase. Dot line: tie line.

As the phase-separated lipid membrane, the composition of POPC/SM/CHOL was selected. This composition is considered to be the main component of the outer leaflet of mammalian cell membranes [29] and shows various phase states as shown in Figure 1 [30]. This is suitable for nanodomain analysis studies, and the composition of (A), (B)-(C)-(D), and (E) were selected as L_o_, (L_o_ + L_d_), and L_d_ phase models, respectively. In addition, the compositions of (B), (C), and (D) are on the same tie line and the L_d_ ratio value in (B), (C), and (D) was 0.25, 0.50, 0.75, respectively, which were calculated by lever rule. 

At first, macroscale lipid membrane analysis was conducted with the solvatochromic fluorescence probe, LipiORDER. Solvatochromic fluorescence probes show the fluorescence peak shift depending on the polarity of the solvent, and thus are effective tools to analyze the lipid membrane polarity (hydration) degree and phase state. Laurdan is one of the most used solvatochromic fluorescence probes, and has been employed for evaluation of phase state (gel phase, liquid phase); Laurdan shows emission peaks at 440 nm (from gel phase) and 490 nm (from liquid phase) when Laurdan is excited at 340–390 nm [31]. However, Laurdan has some problems in the application of cell membrane and SLB analysis; Laurdan is easily photodegraded and its fluorescence property sometimes overlaps with other fluorescence molecules. Therefore, a two-photon microscope must be used in the cell membrane and SLB analysis with Laurdan. On the other hand, LipiORDER, which is a pyrene-based solvatochromic fluorescence dye, is expected to be applied to both cell membranes [32] and SLBs. LipiORDER is also sensitive to polarity (phase state) of the lipid membrane and has high photostability compared to Laurdan in the vesicles. LipiORDER in L_o_ or L_d_ phase emits fluorescence at 510 nm or 575 nm with a 405 nm excitation of light as shown in Supplementary Materials, and its fluorescence intensity ratio (*F*_575_/*F*_510_) can be applied in the phase state analysis [32]. Hence, though SLB analysis with LipiORDER has never been reported, LipiORDER was employed as a phase state indicator for SLB analysis instead of Laurdan, and the characteristic properties of LipiORDER were investigated. 

**Figure 2:**
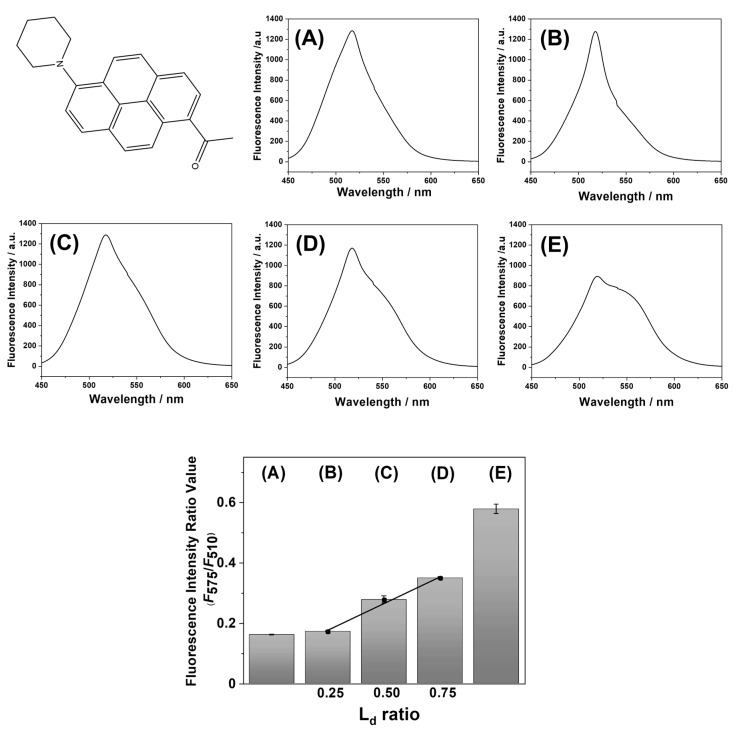
Chemical structure of LipiORDER, fluorescence spectra of LipiORDER in POPC/SM/CHOL SUVs, and fluorescence intensity ratio (*F*_575_/*F*_510_) of (POPC/SM/CHOL) SUVs. Molar ratio (%) of lipid composition (POPC/SM/CHOL): (A) (20/40/40), (B) (37/32/31), (C) (49/29/22), (D) (60/26/14), and (E) (100/0/0). Experimental conditions: see Section 2. Error bar: standard deviation (*N* = 3).

Before the application of LipiORDER for SLB analysis, the phase state analysis of POPC/SM/CHOL small unilamellar vesicles (SUVs) was conducted with LipiORDER. Fluorescence spectra of POPC/SM/CHOL SUVs are shown in Figure 2. POPC/SM/CHOL (20/40/40) and POPC SUVs were selected as model SUVs showing the L_o_ and L_d_ phases, respectively [30]. The ratio values (*F*_575_/*F*_510_) of POPC/SM/CHOL (20/40/40) and POPC/SM/CHOL (100/0/0) SUVs were 0.16 and 0.58, respectively. Furthermore, the ratio values of POPC/SM/CHOL (B: 37/32/31, C: 49/29/22, and D: 60/26/14) SUVs, which are on the same tie line and show the (L_o_ + L_d_) phases (Figure 1) [30], were 0.17, 0.28, and 0.35, respectively. Thus, ratio values increase as the polarity (hydration degree) of the lipid bilayer increases. In addition, linearity (*R*^2^ = 0.99) was found between the ratio values (*F*_575_/*F*_510_) and ratio values of the L_d_ phase area calculated by lever rules (Figure 2). Hence, ratio values (*F*_575_/*F*_510_) can become the index of the L_d_ phase area ratio in phase-separated lipid membranes. In addition, in the (POPC/SM/CHOL) bilayer, SM strongly binds CHOL and forms a L_o_ nanodomain, while POPC forms an L_d_ domain. Therefore, ratio values (*F*_575_/*F*_510_) also showed linearity (*R*^2^ = 0.95) between the ratio (POPC/(SM+CHOL)). In the unknown samples, only this fluorescence method could not correctly characterize the phase state because the threshold values between the phase separation (L_o_ + L_d_) and the homogeneous state (L_o_ or L_d_) could not be determined. However, our results indicate that roughly, the phase state of the target lipid membrane can be categorized on a macroscale with both this LipiORDER probe and the standard model lipid membranes, whose phase state (L_o_ or L_d_ phase) has been reported. In addition, the L_d_ ratio in the phase-separated bilayer can be also estimated with the ratio values (*F*_575_/*F*_510_) of LipiORDER. Thus, the ratio values (*F*_575_/*F*_510_) would be a powerful index for phase analysis of the lipid membrane.

**Figure 3:**
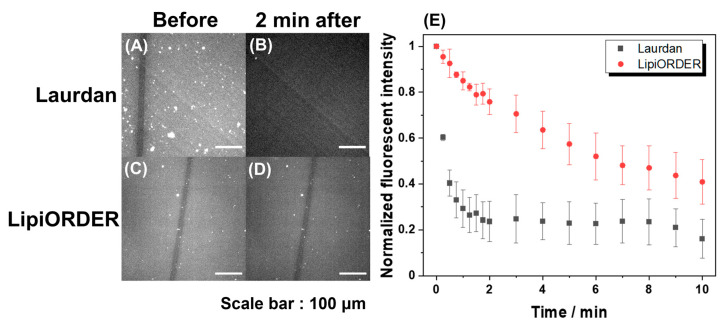
Photostability of Laurdan and LipiORDER in DPPC SLB. Fluorescence microscope images of Laurdan and LipiORDER in DPPC SLB (A,C) before and (B,D) two minutes after photoirradiation. (E) Variation of fluorescence intensity of Laurdan and LipiORDER in DPPC SLB under photoirradiation. Scale bar: 100 μm, filter in Laurdan detection: excitation filters: 340–390 nm, dichroic mirror: 410 nm, barrier filter: 420 nm~. Filter in LipiORDER detection: excitation filters: 389/38 nm, dichroic mirror: 414 nm, barrier filter: 450 nm~. A black line in the images was prepared by scratching DPPC SLB. Error bar: standard deviation (*N* = 6 from different areas and different DPPC SLB).

Subsequently, LipiORDER was applied for planar SLBs with the same composition. The SLBs easily enable membrane analysis in a specific area as well as the whole membrane, which cannot be attained with vesicles. Therefore, much information will be obtained with SLBs. However, generally, the fluorescence analysis of SLB tends to fail because the number of excited molecules in SLB is too low compared to that in vesicle suspension; indeed, the analysis of SLB with Laurdan could not be due to immediate fluorescence quenching (Figure 3). On the other hand, though the fluorescence intensity of LipiORDER also gradually decreased by light irradiation, LipiORDER showed higher photostability and enabled the SLB analysis even though low magnification lenses were used. In addition, LipiORDER could reduce the amount of usage to less than one-third of Laurdan in SLB analysis, which means that LipiORDER allows SLB analysis with less damage on SLB. From this result, it is clear that LipiORDER can be applied for SLB macroscale analysis 

**Figure 4:**
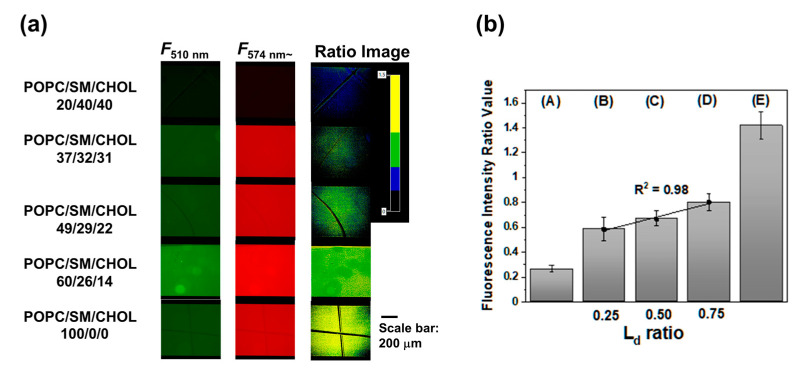
(**a**) Fluorescence microscope images and ratio images of (POPC/SM/CHOL) SLBs and (**b**) ratio values. The black line was prepared by scratching the SLB. Error bar: standard deviation (*N* > 5 from different areas and different SLBs). Molar ratio (%) of lipid composition (POPC/SM/CHOL): (A) (20/40/40), (B) (37/32/31), (C) (49/29/22), (D) (60/26/14), and (E) (100/0/0).

As the applicability of LipiORDER in SLBs was proved, phase state analysis of SLBs was conducted by fluorescence ratio images with a simultaneous dual wavelength system. This simultaneous dual-wavelength system would be essential for robust phase state analysis even though LipiORDER shows higher photostability. Figure 4 shows fluorescence and fluorescence ratio images of POPC/SM/CHOL SLBs. Similar to SUV experiments, SLBs were prepared with POPC/SM/CHOL (20/40/40) or POPC/SM/CHOL (100/0/0), which are the standard models of L_o_ or L_d_ phase membrane, respectively. POPC/SM/CHOL (20/40/40) or POPC/SM/CHOL (100/0/0) SLBs showed strong fluorescence intensity from 510/84 nm or 574 nm~ and its mean ratio values (*R* = *F*_574~_/*F*_510_) in several areas were 0.26 and 1.4, respectively. Furthermore, SLBs prepared from POPC/SM/CHOL (37/32/31, 49/29/22, and 60/26/14) showed that the fluorescence derived from both the L_o_ and L_d_ phase was observed through a 510/84 nm and 574 nm~ filter. 

The mean ratio values were higher than those of the POPC/SM/CHOL (20/40/40) SLBs and lower than those of the POPC SLBs, which is the same result as that of vesicles and implies that SLBs from POPC/SM/CHOL (37/32/31, 49/29/22, and 60/26/14) show phase separation (L_o_ + L_d_). As SLB is on a hydrated layer between solid substrates [33], the phase state of SLB and domain sizes would be affected by the curvature, surface roughness, charge, etc., of the substrate in some cases [34]. In this case, the phase state analysis with vesicles and SLBs indicates that the phase state in POPC/SM/CHOL SLBs corresponded with those in vesicles because of the following reasons: Ratio values (*F*_574~_/*F*_510_) showed a linear correlation (*R^2^* = 0.98) between ratio values (L_d_ phase area) calculated by lever rules as well as the vesicle case. 

On the other hand, in this composition, domain morphology could not be observed because the size of the domain is on a nanometer scale as shown in SPM’s data later. However, the domains in SLBs can be observed in some compositions with the fluorescence microscope. In such a case, the ratio images will clearly visualize the phase state of domains and be a more powerful analytical method. Anyway, it can be concluded that LipiORDER can be applied for phase state analysis of SLB on the macroscale. It requires a small amount of usage, and does not need an expensive two-photon microscope and high magnification lenses. Thus, LipiORDER enables researchers to conduct the phase state analysis of SLB on a macroscale easily.

**Figure 5:**
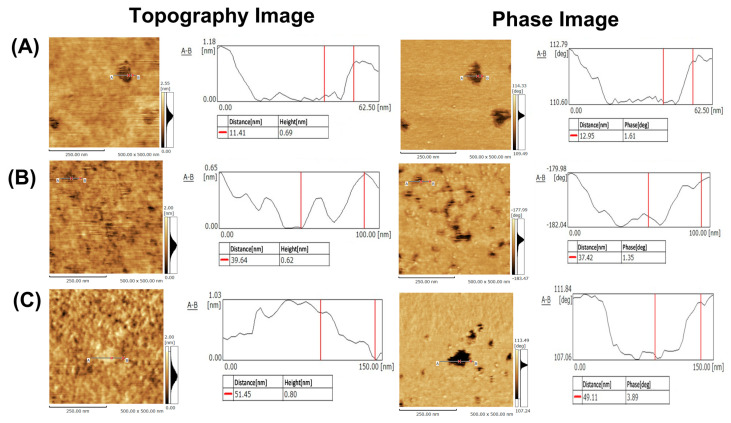
Topography and phase images of (POPC/SM/CHOL) SLBs by SPM. The molar ratio (%) of lipid composition (POPC/SM/CHOL): (A) 37/32/31, (B) 49/29/22, and (C) 60/26/14. Experimental conditions: see Section 2.

Finally, microscale analysis by SPM was conducted. The resolution of SPM is reported to be on the order of 0.1 nm in the height direction, and 1–10 nm in the lateral direction [35]. This superior performance is suitable for nanodomain analysis in planar SLBs because the height difference between the nanodomain and its surroundings would be ca. 0.5–1.5 nm in planar SLB [23,24]. However, it is required to consider the measurement conditions for the best performance of soft lipid membrane analysis, e.g., the measurement mode, spring constant of the cantilever, force applied to the cantilever, size of the cantilever, and resonance frequency [36]. In addition to SPM conditions, the preparation conditions for SLB must also be paid attention to; it has been reported that the observed membrane state varies depending on the substrate of the lipid membrane and the preparation conditions of the SLB [34]. This is because SPM has atomic resolution and SLB is also affected by the surface roughness of the substrate itself and the preparation conditions. Therefore, we carefully selected the SPM conditions and conducted careful pretreatment of the glass substrate. SLBs from POPC/SM/CHOL (37/32/31, 49/29/22, and 60/26/14) were selected as model samples for SPM analysis because these SLBs show phase separation. In POPC/SM/CHOL (37/32/31 and 49/29/22) SLBs, nanodomains were observed, whose height was lower than that of the surrounding (Figure 5A,B).

On the other hand, POPC/SM/CHOL (60/26/14) SLB showed nanodomains, whose height was higher than that of the surrounding (Figure 5C). In both cases, the height difference was 0.9 ± 0.28 nm. Generally, the thickness of lipid bilayers is ca. 5 nm, and thus nanoregions in POPC/SM/CHOL (37/32/31 and 49/29/22) SLBs are never defects, which is also confirmed by phase mode imaging (as stated below); if this nanoregion is the defect, the height difference becomes ca. 5 nm. Furthermore, the size of the domains in each composition was on the order of several tens of nanometers. As aforementioned, the state of SLB is affected by the substrate itself, substrate roughness, and SLB preparation conditions. In our case, after liposome adsorption, rapture, and SLB formation, SLB was immediately cooled down to 25 °C. This condition may hamper the formation of large-sized nanodomains (over 100 nm in size) and result in several tens to hundreds of nanometer-sized nanodomains. In cell membranes, molecules immobilized by the actin framework prevent the macroscopic phase separation of lipid membranes, resulting in the formation of nanodomains [37]. Therefore, our conditions might be useful for the preparation of cell-imitated SLB with several tens of nanometer domains. In addition to size and height differences, Figure 5C also indicates that the state of phase separation was reversed compared to that of Figure 5A,B, in which the domain is protruded or depressed. Generally, the height of the L_o_ domain is higher than that of the L_d_ domain because lipid molecules are tightly packed in the L_o_ domain and loosely packed in the L_d_ domain. Therefore, it can be concluded that the L_d_ nanodomain (island) and the L_o_ nanodomain (island) were formed in Figure 5A,B and Figure 5C, respectively. This phase inversion is related to complex factors, and thus cannot be attributed to only some factors, but would be due to the ratio of POPC/(SM and CHOL) [38]. SM strongly binds CHOL and forms L_o_ nanodomains, while POPC forms L_d_ domains. Therefore, this is a rough discussion, but the decreasing of (SM+CHOL) might cause an inversion of the phase state. In conclusion, we successfully proved that SPM is an effective analysis method for domain size, type of domain, and domain morphology, which are not elucidated by fluorescence analysis. 

The visualization of nanodomains was further attempted by phase images. Phase images are one of the qualitative analysis methods of SPM and have been frequently employed to visualize microphase separation in polymer films [39]. In phase imaging, the difference in surface properties (viscoelasticity, adhesion, etc.) is visualized mainly with the phase delay between the driving signal and the output signal. Therefore, this phase image was applied for the analysis of nanodomains in SLBs. Phase images of POPC/SM/CHOL SLBs are shown in Figure 5A–C. These images clearly show that there are two states with different physical properties in these SLBs. Furthermore, compared with the topography image, two regions could be clearly observed due to the difference in physical properties. Thus, phase state and nanodomain (shape, type, size) could be clearly visualized due to different surface properties and height differences by both phase images and topography. Unfortunately, though this SPM performance cannot conduct surface properties analysis of both domains and domain outer regions, other types of SPM could analyze the properties and visualize nanodomains. One of the examples is the viscoelastic analysis, which reported that the L_o_ domain would show a higher Young modulus, while the L_d_ domain is lower than that [40]. Thus, SPM would become a powerful analysis method for both nanodomains and their surrounding analyses. 

From the above results, we successfully demonstrated that our improved methods could analyze the phase-separated lipid membrane. Ratio analysis with LipiORDER was an effective analysis method for the phase state of the lipid membrane, especially the planar lipid bilayer membrane. Topography with a scanning probe microscope enabled the analysis of specific nanodomain properties, such as size, roughness, shape, and type of nanodomain. In addition, the phase mode of SPM clearly visualized nanodomain by properties difference, even though nanodomain roughness and size are too small. Thus, the combination of fluorescence analysis with LipiORDER and SPM (topography and phase images) enables phase state analysis of lipid membranes on the macro and microscale, and this would accelerate the research of lipid membranes, including real cell membranes. 

## 4. Conclusions

We successfully proved the utility of the solvatochromic fluorescence probe (LipiORDER) and SPM in the phase state study of the lipid membrane, especially of SLB. This probe roughly informs the phase state of the SLB on a macroscale with less damage and without an expensive two-photon microscope. In addition, the topography images and phase mode of the SPM could detect a few nanometer domains in the SLB and visualize them on the microscale. Therefore, this combination method would be a powerful tool for the phase state (raft domain) analysis on the micro and macro scale even though the nanodomain size is a few nanometers.

## Supplementary Materials

The following supporting information can be downloaded at: https://www.mdpi.com/article/10.3390/membranes12080770/s1, Figure S1: Second derivative fluorescence spectrum of LipiORDER in (POPC/SM/CHOL) SUVs.
