# Supplementary material for: Characterization of Phase Separated Planar Lipid Bilayer Membrane by Fluorescence Ratio Imaging and Scanning Probe Microscope
Source: Membranes (Basel). 2022 Aug 9;12(8):770. doi: 10.3390/membranes12080770 (PMC9415343; doi:10.3390/membranes12080770)
Supplement: Supplementary file 1 [file membranes-12-00770-s001.zip › membranes-1848169-supplementary.pdf]

*Supplementary Material*

# Characterization of Phase Separated Planar Lipid Bilayer Membrane by Fluorescence Ratio Imaging and Scanning Probe Microscope

**Yukihiro Okamoto[1]*, Kaito Hamaguchi[1], Mayo Watanabe[1], Nozomi Watanabe[1] and Hiroshi Umakoshi[1]**

Division of Chemical Engineering, Graduate School of Engineering Science, Osaka University, 1-3 Machikaneyama-cho, Toyonaka, Osaka 560-8531

* Correspondence: okamoto.yukihiro.es@osaka-u.ac.jp

The two states of LipiORDER in (POPC/SM/CHOL) SUVs and these peak wavelengths were confirmed with the second derivative fluorescence spectra. The second derivative fluorescence spectra in Supporting Figure 1 were obtained by the Origin graph software (Lightstone, Tokyo, Japan) under following conditions: Savitzky-Golay algorithm, a quadratic polynomial of 15 points, and 0.5 nm interval. The spectra show that the two peaks could be found at 505~515 nm and 560~585 nm ($L_d$ phase) in (B) (37/32/31), (C) (49/29/22), (D) (60/26/14) and (E) (100/0/0), and only one peak in (A) (POPC/SM/CHOL: 20/40/40), which is from $L_o$ phase. Hence, the fluorescence ratio value derived from $L_o$ and $L_d$ phase is effective for phase state analysis.

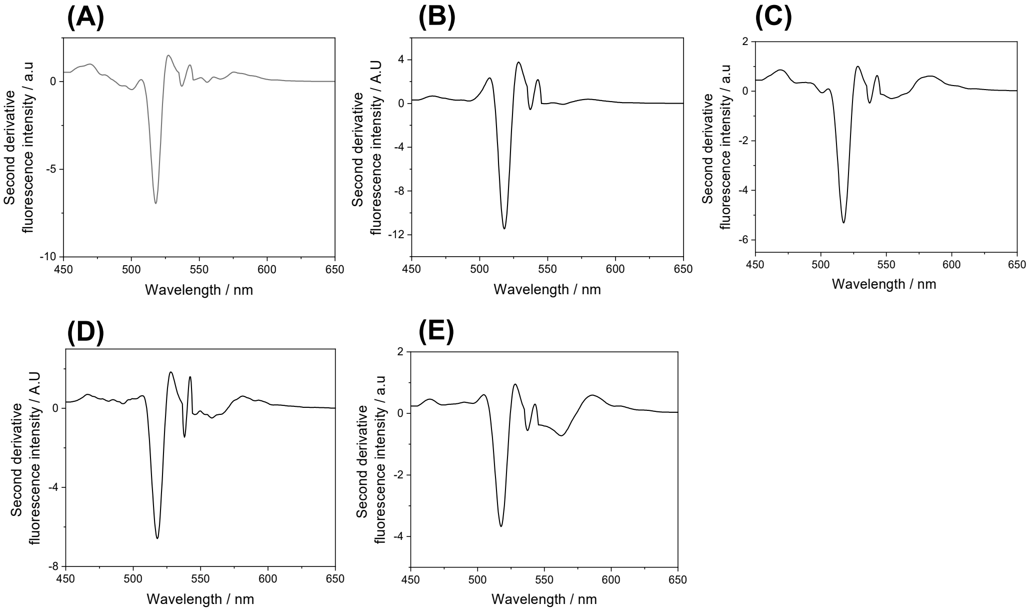


**Supporting Figure 1.** Second derivative fluorescence spectrum of LipiORDER in (POPC/SM/CHOL) SUVs. Molar ratio (%) of lipid composition (POPC/SM/CHOL): (A) (20/40/40), (B) (37/32/31), (C) (49/29/22), (D) (60/26/14) and (E) (100/0/0).
